# Comparative protective effects of rosuvastatin and ramipril against doxorubicin-induced testicular toxicity in rats: A multimodal evaluation of oxidative stress and reproductive parameters

**DOI:** 10.14202/vetworld.2025.2264-2272

**Published:** 2025-08-09

**Authors:** B. Anisha, Shreya Hegde, Shivaprakash Gangachannaiah, Bharti Chogtu, Guruprasad Kalthur, Sneha G. Kalthur

**Affiliations:** 1 Department of Pharmacology, Kasturba Medical College, Manipal, Manipal Academy of Higher Education, Manipal, Karnataka, India; 2 Division of Reproductive Biology, Department of Reproductive Science, Kasturba Medical College, Manipal, Manipal Academy of Higher Education, Manipal, Karnataka, India; 3 Department of Research and Innovation, Sree Mookambika Institutes, Padanilam, Kulasekharam-629161; 4 Department of Anatomy, Kasturba Medical College, Manipal, Manipal Academy of Higher Education, Manipal, Karnataka, India; 5 Department of Anatomy, Sree Mookambika Institutes, Padanilam, Kulasekharam, Tamil Nadu, India

**Keywords:** doxorubicin, testicular toxicity, oxidative stress, ramipril, rats, rosuvastatin, sperm motility

## Abstract

**Background and Aim:**

Doxorubicin, a widely used chemotherapeutic agent, is associated with reproductive toxicity due to its induction of oxidative stress and testicular damage. Emerging evidence suggests that rosuvastatin and ramipril may possess antioxidant and cytoprotective properties beyond their conventional uses. However, their comparative efficacy in preventing doxorubicin-induced testicular toxicity remains unclear. This study aimed to evaluate and compare the protective effects of rosuvastatin and ramipril on testicular function, oxidative stress markers, and reproductive outcomes in a rat model of doxorubicin-induced testicular toxicity.

**Materials and Methods:**

Twenty-four male: Wistar rats were randomly allocated into four groups: Control, doxorubicin-only, rosuvastatin + doxorubicin, and ramipril + doxorubicin. Doxorubicin (5 mg/kg, intraperitoneal) was administered on days 7, 14, and 21, while rosuvastatin or ramipril (5 mg/kg/day, oral) was given for 21 days. On day 45, evaluations included testicular index, sperm count and motility, serum testosterone levels, oxidative stress markers (malondialdehyde [MDA], nitric oxide [NO], glutathione [GSH]), and histopathological analysis using Johnsen scoring.

**Results:**

Both rosuvastatin and ramipril significantly restored the testicular index compared to the doxorubicin group (p < 0.05). Ramipril markedly increased serum testosterone, GSH, and NO levels while reducing MDA. Sperm motility and count showed partial improvement, notably in the ramipril group. Histopathological alterations were attenuated in both treatment groups, with improved Johnsen scores and reduced architectural disruption.

**Conclusion:**

Ramipril and rosuvastatin mitigate doxorubicin-induced testicular toxicity through antioxidant mechanisms. Ramipril demonstrated superior efficacy in preserving reproductive hormone levels and sperm function. These findings highlight its potential as a fertility-protective agent during chemotherapy. Further long-term and mechanistic studies are warranted.

## INTRODUCTION

Doxorubicin, a widely employed anthracycline chemotherapeutic agent, is known to induce significant reproductive toxicity in both male and female subjects. In males, its deleterious effects stem from disruption of Leydig and Sertoli cell function and interference with testicular lipid metabolism. A central mechanism underlying this toxicity is the induction of oxidative stress, primarily mediated by the downregulation of critical antioxidant enzymes, including superoxide dismutase, catalase, and glutathione (GSH) peroxidase. Furthermore, doxorubicin inhibits topoisomerase II acti-vity, leading to excessive generation of reactive oxygen species (ROS) and consequent tissue damage [1]. The strong association between ROS and testicular injury has made doxorubicin-induced toxicity a widely acce-pted experimental model for evaluating the efficacy of potential protective agents.

Rosuvastatin, a potent 3-hydroxy-3-methylglutaryl-CoA reductase inhibitor primarily used to manage hyperlipidemia, exhibits additional pharmacological benefits beyond lipid lowering. Notably, rosuvastatin has demonstrated anti-inflammatory and antifibrotic acti-vities and has been shown to modulate oxidative stress and topoisomerase II activity in models of doxorubicin-induced cardiotoxicity [2]. Other statins, including fluv-astatin and atorvastatin, similarly exert antioxidant effects by scavenging ROS and mitigating cellular dam-age [3, 4]. These agents also enhance endogenous anti-oxidant defenses by upregulating the expression and activity of catalase, superoxide dismutase, and GSH per-oxidase [5].

Ramipril, an angiotensin-converting enzyme inhibitor (ACEI), is widely used in the management of hypertension and cardiovascular disorders. The renin–angiotensin system (RAS), which ramipril modulates, plays a vital role in male reproductive physiology, part-icularly in regulating sperm function and Leydig cell activity [6]. Interestingly, while RAS inhibition may dec-rease sperm motility, it does not appear to affect sperm count or morphology significantly [7].

Although both statins and ACEIs have shown protective effects against testicular damage in various preclinical models, their comparative efficacy under identical pathological conditions has not been thor-oughly investigated. Existing studies have explored the individual roles of agents such as atorvastatin, fluva-statin, and captopril in mitigating oxidative stress and preserving testicular architecture [4, 5, 8]. However, no direct comparative analysis has been conducted to evaluate rosuvastatin and ramipril within a uniform exp-erimental framework.

While doxorubicin-induced testicular toxicity is well-established as a model for evaluating antioxidant and cytoprotective agents, limited research exists on the comparative efficacy of pharmacologically distinct cardioprotective agents in this context. Both statins and ACEIs have demonstrated individual potential in ameliorating oxidative stress and preserving testicular structure and function. Specifically, studies involving fluvastatin, atorvastatin, and captopril have reported improvements in antioxidant enzyme levels, histological preservation, and hormonal balance in testicular tiss-ues subjected to oxidative insults. However, despite rosuvastatin’s known pleiotropic effects and ramipril’s modulation of the RAS – both of which are implicated in testicular physiology – no studies to date have directly compared their protective capabilities within the same experimental model of doxorubicin-induced testicular damage. Furthermore, the mechanistic underpinnings of how these agents influence key reproductive endpoints, such as sperm motility, testos-terone synthesis, and oxidative stress markers, remain underexplored in a side-by-side evaluation. This presents a critical knowledge gap, particularly in the context of developing adjunctive therapies to mitigate chemotherapy-induced reproductive dysfunction.

The present study aimed to systematically evaluate and compare the protective effects of rosuvastatin and ramipril on doxorubicin-induced testicular toxicity in a rat model. Specifically, the study assessed their impact on testicular index, sperm count and motility, serum testosterone concentrations, and key oxidative stress markers, including malondialde-hyde (MDA), nitric oxide (NO), and GSH. In addition, histopathological changes were analyzed using the Johnsen scoring system to assess the extent of structural preservation in the testicular tissue. By integrating biochemical, functional, and morphological assess-ments, this study seeks to elucidate the relative efficacy of rosuvastatin and ramipril in mitigating reproductive toxicity and to identify the more promising candidate for further translational research in the context of male fertility preservation during chemotherapy.

## MATERIALS AND METHODS

### Ethical approval

The experimental protocol was approved by the Institutional Animal Ethics Committee of Kast-urba Medical College, Manipal (Approval No: IAEC/KMC/91/2022, dated August 12, 2022). All procedures were conducted in accordance with the Animal Rese-arch: Reporting of *In Vivo* Experiments (ARRIVE) guidelines. A total of 24 healthy male Wistar rats (8 weeks old, weighing 200–250 g) were procured from the Central Animal Facility. Animals were housed in sterile polypropylene cages under standard laboratory conditions (12-h light/dark cycle, 25°C ± 3°C, 50% relative humidity), with *ad libitum* access to pelleted feed and water.

### Study period and location

The study was conducted from September 2022 to August 2023 in Department of Pharmacology and Division of Reproductive Science, Kasturba Medical College, Manipal.

### Experimental design and grouping

Rats were randomly assigned to four groups (n = 6/group) using a computer-generated randomization method:

- Group 1 (control): Received no treatment
- Group 2 (doxorubicin): Received doxorubicin only
- Group 3 (rosuvastatin + doxorubicin): Received rosuvastatin and doxorubicin
- Group 4 (ramipril + doxorubicin): Received ramipril and doxorubicin.

Doxorubicin (ADRIM, Medas Pharma, Gujarat, India) (5 mg/kg, intraperitoneally) was administered on days 7, 14, and 21. Rosuvastatin (Roseday, USV Pvt. Ltd, Mumbai, India) or ramipril (Ramistar, Lupin Ltd, Mumbai, India), both (5 mg/kg/day, orally), were administered once daily for 21 consecutive days. All outcome assessors were blinded to treatment allocation to minimize bias. Dosages were selected based on previous studies by Aykan [7], Sherif and Sarhan [9], and Lee *et al*. [10].

### Drug preparation and administration

Doxorubicin was administered intraperitoneally at a dose of 5 mg/kg on days 7, 14, and 21. Rosuva-statin and ramipril were each administered orally at 5 mg/kg/day for 21 days. Drugs were suspended in freshly prepared distilled water before administration.

### Sample collection

On day 45, animals were euthanized through intra-peritoneal overdose of ketamine (150 mg/kg). Blood samples were collected from the retro-orbital plexus between 9:00 and 10:00 AM, centrifuged at 600 × *g* for 8 min, and serum was stored at −80°C for testosterone analysis. One testis from each rat was fixed in 10% neutral-buffered formalin for histological evaluation, while the contralateral testis was snap-frozen in liquid nitrogen and stored at −80°C for biochemical assays. Testes were later homogenized in phosphate-buffered saline (PBS), and the supernatant was used for oxidative stress assessments.

### Testicular index measurement

Body weights were recorded on days 0 and 45. At sacrifice, the testes were weighed, and the testicular index (TI) was calculated using the following formula [9]:

TI = (Testis weight on day 45/Body weight on day 45) × 100

### Sperm analysis

The cauda epididymis was excised, and sper-matozoa were released into pre-warmed saline cont-aining 0.1% bovine serum albumin. The suspension was diluted 1:10 in PBS. Sperm count was evaluated using a Neubauer hemocytometer (Rohem, Germany). Sperm motility was assessed under phase-contrast microscopy at 37°C within 10 min of collection, and the percentage of progressively motile sperm was recorded [10].

### Serum testosterone estimation

Serum testosterone levels were quantified using a commercially available ELISA kit (Calbiotech Inc., USA), following the manufacturer’s protocol.

### Assessment of oxidative stress markers

Testicular tissue homogenates were analyzed for MDA, NO, and GSH levels:

- MDA (µM/L): Assessed using the thiobarbituric acid reaction method [11–13].
- NO (µM/mg protein): Quantified using the Griess reagent method [14].
- GSH (mg/mg protein): Estimated using Ellman’s reagent according to standard protocols [15].

All biochemical assays were performed in duplicate using reagents from Sigma-Aldrich (St. Louis, MO, USA).

### Histopathological evaluation

Testicular tissues were fixed in 10% neutral-buffered formalin for 48 h, embedded in paraffin, and sectioned at 5 μm thickness. Hematoxylin and eosin staining was performed according to the method of Bancroft and Stevens. Histological damage was assessed using Johnsen’s scoring system (scale 1–10) on 10 randomly selected seminiferous tubules per animal. Two independent pathologists, blinded to the treatment groups, evaluated all sections [7].

### Statistical analysis

Normality of data distribution was tested using the Shapiro–Wilk test. Group comparisons were made using one-way analysis of variance, followed by Tukey’s *post hoc* test (GraphPad Prism v8.0.1, GraphPad Software, USA). All values are expressed as mean ± stan-dard deviation. A p < 0.05 was considered statistically significant.

## RESULTS

### Testicular index

**Figure 1:**
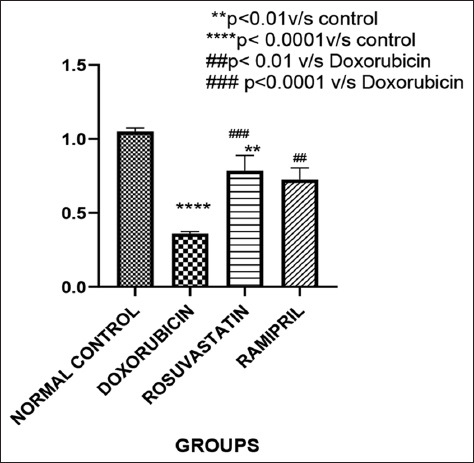
Testicular indices of the different groups of patients.

As illustrated in Figure 1, doxorubicin administr-ation (Group 2) resulted in a significant reduction in testicular index compared to the control group (Group 1) (p < 0.05). Both treatment groups – rosuvastatin (Group 3) and ramipril (Group 4) – demonstrated signi-ficant restoration of testicular index relative to the doxo-rubicin-only group (p < 0.05), indicating a protective effect of both interventions on testicular mass.

### Sperm count and motility

**Figure 2:**
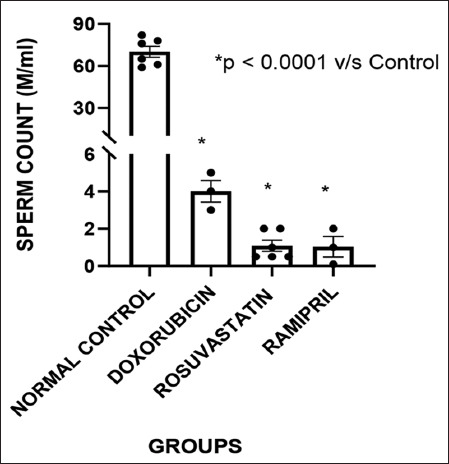
Sperm counts of different groups.

Sperm parameters across all experimental groups are shown in Figure 2. A substantial decline in sperm count was observed in the doxorubicin group (p < 0.05 vs. control). Sperm counts did not show any improvement in the rosuvastatin and ramipril groups. A similar trend was observed in sperm motility: Doxorubicin significantly reduced motility, whereas partial restoration was observed in both treatment groups, with the ramipril group exhibiting slightly better recovery.

### Serum testosterone levels

**Figure 3:**
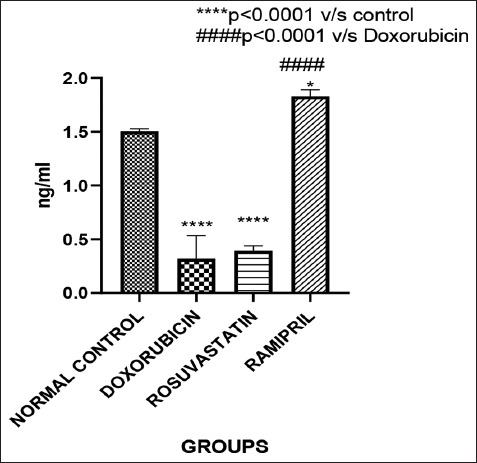
Serum testosterone levels of different groups.

Group-wise serum testosterone levels are pres-ented in Figure 3. Both the doxorubicin (Group 2) and rosuvastatin (Group 3) groups showed a significant reduction in testosterone concentrations compared to the control (p < 0.05). Interestingly, the ramipril group (Group 4) demonstrated a significant elevation in testosterone levels relative to both the doxorubicin and control groups (p < 0.05), suggesting a hormone-preserving effect.

### Oxidative stress markers

#### MDA

**Figure 4:**
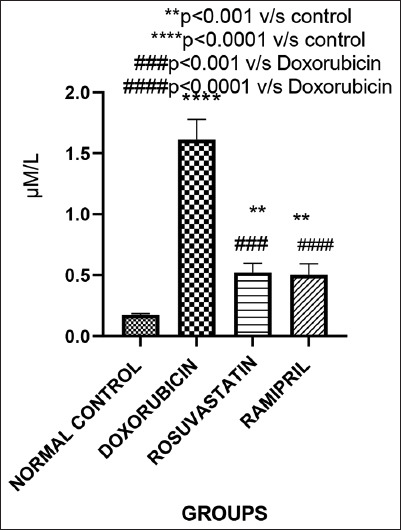
Malondialdehyde levels across the groups.

As shown in Figure 4, MDA levels – indicative of lipid peroxidation and oxidative damage – were sig-nificantly elevated in the doxorubicin group. Both rosuvastatin and ramipril treatments significantly reduced MDA concentrations (p < 0.05 vs. Group 2), reflecting their antioxidative effects.

#### NO

**Figure 5:**
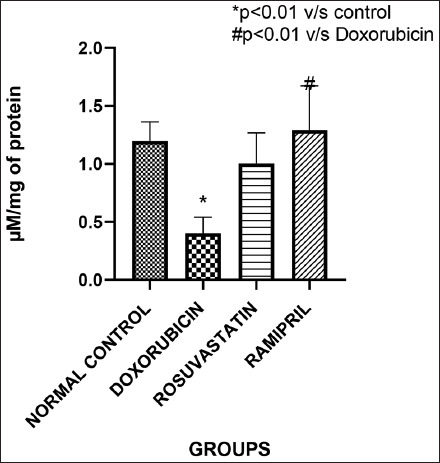
Nitric oxide levels across the groups.

Figure 5 illustrates NO levels across groups. A significant increase in NO was observed exclusively in the ramipril group (p < 0.05 vs. group 2), whereas the rosuvastatin group showed a non-significant increase compared to the doxorubicin group.

#### GSH

**Figure 6:**
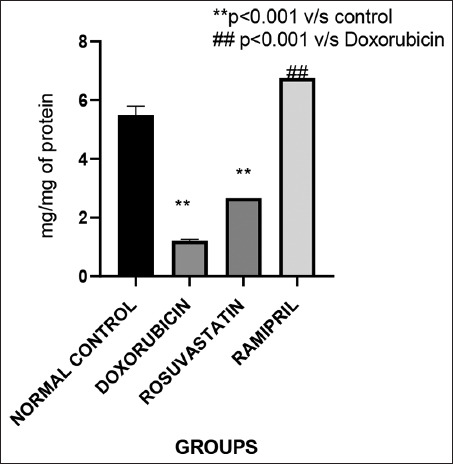
Glutathione levels across the groups.

Figure 6 displays testicular GSH concentrations. While the rosuvastatin group showed a marginal increase, the ramipril group exhibited a statistically significant elevation in GSH levels compared to the doxorubicin group, suggesting superior restoration of antioxidant defenses.

### Histopathological findings and Johnsen scoring

**Figure 7:**
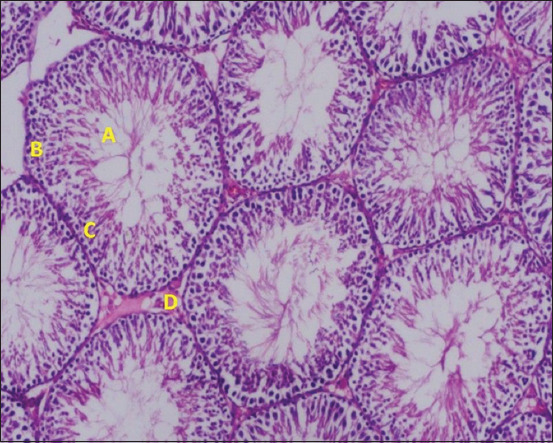
Histopathological findings in Group 1 (normal control). (A) Spermatocyte, (B) basement membrane, (Cc) intact tubular epithelium, and (D) Leydig cells.

**Figure 8:**
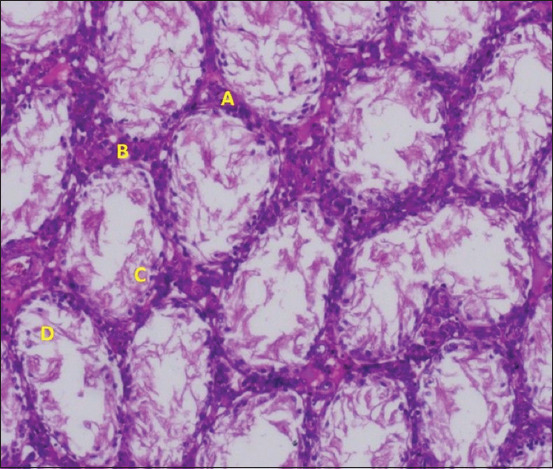
Histopathological findings in the 2-doxorubicin group. (A) Thickening in the basement membrane (B) inte-rstitial fibrosis, (C) desquamated cells, and (D) sloughing of the epithelium.

**Figure 9:**
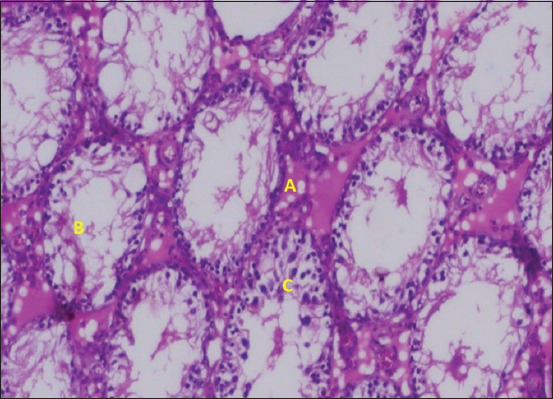
Histopathological findings in group 3. (A) Vacu-olization in interstitial tissue, (B) desquamated cells, and (C) disrupted tubular epithelium.

**Figure 10:**
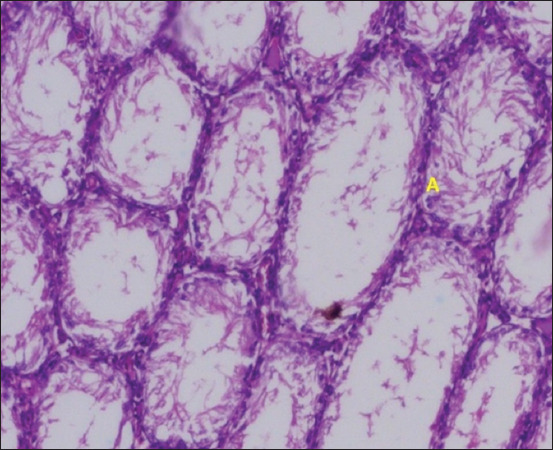
Histopathological findings in group 4 (Ramipril). (A) Tubular lumen with few spermatocytes.

Histopathological outcomes are summarized in Table 1 and depicted in Figures 7–10. The control group (Group 1) exhibited normal testicular architecture with a Johnsen score of 10. In contrast, all experim-ental groups (Groups 2–4) had a Johnsen score of 3. The doxorubicin group (Group 2) showed severe stru-ctural abnormalities, including disrupted basement membranes, desquamated epithelial cells, vacuoli-zation of tubules and interstitial tissue, and interstitial fibrosis.

**Table 1:** Histopathological analysis using Johnsen’s criteria for different groups.

Testicular structures	Normal control	Doxorubicin	Rosuvastatin	Ramipril
Tunica albuginea	Present	Thickened subcapsular hemorrhage	Thickened subcapsular hemorrhage	Thickened subcapsular hemorrhage
Basement membrane of the tubule	Present	Tubules small in diameter with BM (basement membrane) thickening	++	+
Spermatogonia germ cell count	Normal count with intact tubular epithelium	disrupted basement membrane and tubular epithelium	++ disrupted tubular epithelium	disrupted tubular epithelium+
Spermatocytic arrest	Absent	Present	Present	Present
Peritubular or interstitial fibrosis	Absent	Present with inflammatory cells ++	Present with inflammatory cells +	Present with inflammatory cells +
Sloughing or disorganization	Absent	disordered spermatogenesis	disordered spermatogenesis	disordered spermatogenesis
Vacuolization in the tubule	Absent	Present	Absent	Absent
Vacuolization in the interstitial tissue	Absent	Present ++	++	+
Tubular lumen	Sperm	Desquamated cells	Desquamated cells	Desquamated cells
Multinucleated giant cells	Absent	Absent	Absent	Absent
Vascular congestion or dilatation	Absent	Increased vascularity ++	++	+
Sertoli cell	Normal	Few	Few	Few
Leydig cell	Normal	Normal	Normal	Normal
Johnsen criteria	10	3	3	3

Treatment with rosuvastatin (Group 3) and ramipril (Group 4) partially alleviated these changes. Both groups demonstrated reduced interstitial fibrosis and absence of tubular vacuolization. While complete architectural recovery was not achieved, the histological damage was notably milder compared to the doxorubicin-only group, indicating partial testicular protection.

## DISCUSSION

### Validation of the experimental model and study objectives

This study utilized a well-established model of doxorubicin-induced testicular toxicity to investigate the protective potential of rosuvastatin and ramipril. The efficacy of these agents was systematically evaluated through functional (testicular index and sperm quality), hormonal (testosterone levels), biochemical (oxid-ative stress markers), and histological (Johnsen score) parameters.

### Effect of doxorubicin and interventions on body and testicular indices

Doxorubicin administration led to a marked decline in body weight, in line with previous study by Lee *et al*. [10] demonstrating weight loss in rats following single or repeated intraperitoneal doses. In addition, there was a significant reduction in testicular weight and testicular index, consistent with earlier findings on doxorubicin-induced testicular atrophy [16]. Interestingly, while rosuvastatin also resulted in a reduced testicular index compared to control animals, similar observations were not reported in prior studies using pravastatin [16]. Notably, ramipril treatment improved the testicular index relative to the doxorubicin group. This may be attributed to its regulatory influence on peroxisome proliferator-activated receptor gamma (PPAR-γ), which is known for its anti-inflammatory and antiproliferative roles in models of anthracycline-induced organ damage [17, 18].

### Impact on sperm count and motility

Doxorubicin significantly impaired sperm count and motility, corroborating earlier preclinical reports [10]. Prior evidence suggesting ACE inhibitors can improve certain sperm parameters in toxic injury models [19] was not observed in present study with respect to sperm count. In addition, a previous study by Villela e Silva *et al*. [20] combining darbepoetin with ramipril further supported its protective reproductive effects. This improvement is likely due to ramipril’s antioxidant activity. Rosuvastatin had negligible effects on sperm motility in this study, which aligns with prior observations where chronic statin administration did not reverse sperm functional deficits.

### Modulation of serum testosterone levels

The study revealed a significant decline in serum testosterone concentrations in both the doxor-ubicin and rosuvastatin groups. These results support earlier findings that statins may suppress testoste-rone biosynthesis, potentially through inhibition of cholesterol-derived steroidogenesis [7]. In contrast, ramipril significantly elevated testosterone levels relative to both the doxorubicin and control groups. This effect is supported by prior work indicating that ACE inhibitors, such as captopril, can restore testosterone levels in toxicological injury models [8]. Likewise, angiotensin receptor blockers like candesartan have demonstrated similar effects in cisplatin-induced testi-cular toxicity [9].

### Antioxidant effects and lipid peroxidation (MDA levels)

Doxorubicin treatment significantly elevated MDA levels, indicative of increased lipid peroxidation and oxidative stress – a well-documented effect in testicular toxicity models [16, 21]. Both rosuvastatin and ramipril significantly reduced MDA concentrations in this study. This is consistent with prior findings showing fluvastatin’s antioxidative efficacy in comparable sett-ings [22]. Moreover, both ACE inhibitors and ARBs have been reported to suppress MDA levels while enhancing antioxidant defenses in testicular injury models [8].

### Influence on NO levels

Only ramipril significantly increased NO levels, while rosuvastatin caused a mild, non-significant elev-ation. This observation contrasts with prior studies in which captopril and telmisartan decreased NO prod-uction, likely through suppression of NO synthase activity [8]. The increase in NO levels with ramipril in this study may reflect a compensatory antioxidative resp-onse. Statins are known to stimulate endothelial NO synthase activity through Rho/ROCK (Rho-associated protein kinase) pathway inhibition and AMPK (AMP-activated protein kinase) activation, although this effect was not strongly evident here [23].

### GSH restoration

Ramipril significantly elevated testicular GSH concentrations, while rosuvastatin produced only a marginal, statistically insignificant increase. These resu-lts are consistent with previous study by Zinellu and Mangoni [24] demonstrating that statins can enhance antioxidant enzyme activities, including GSH peroxidase and superoxide dismutase, across multiple tissue types. Atorvastatin, in particular, has been shown to elevate GSH levels in cadmium-induced hepatic injury models [25]. Similarly, ramipril has been reported to restore GSH levels in ovarian tissues subjected to ischemia-reperfusion injury [26], which supports the robust antioxidant effect observed in the present study.

### Histopathological outcomes and tissue protection

Histological analysis confirmed extensive tes-ticular damage in doxorubicin-treated animals, including thickened basement membranes, epithelial desquamation, vacuolization, and interstitial fibrosis. These structural disruptions mirror previously reported histopathological changes associated with anthracycline toxicity [9]. Both rosuvastatin and ramipril ameliorated these pathological alterations to a moderate extent. The protective effects of statins are likely linked to their antioxidant, anti-inflammatory, and anti-apoptotic properties. Meanwhile, ramipril’s efficacy may stem from its ability to reduce oxidative damage and inhibit inflammatory pathways such as nuclear factor-kappa B (NF-κB) signaling.

## CONCLUSION

In this experimental model of doxorubicin-induced testicular toxicity, both rosuvastatin and ramipril demonstrated significant protective effects, as evidenced by the restoration of testicular index, reduction in MDA levels, and improved histoarchitecture of the testes. Ramipril showed superior efficacy by significantly elevating serum testosterone, increasing GSH and NO levels, and partially restoring sperm motility, suggesting a more comprehensive protective role compared with rosuvastatin.

From a practical perspective, these findings underscore the potential of ACE inhibitors, such as ramipril, as adjunctive therapies to counteract repro-ductive side effects associated with doxorubicin. This is particularly relevant for male patients undergoing cancer treatment, where fertility preservation is a growing clinical concern.

A key strength of this study lies in its comparative design, which allows for the direct assessment of two pharmacologically distinct agents under identical experimental conditions. In addition, the evaluation encompassed functional, biochemical, and histological endpoints, offering a multidimensional understanding of testicular protection mechanisms.

However, this study has limitations. The study was confined to a short-term model, lacked assessments of fertility outcomes, and did not explore the molecular signaling pathways underlying the observed effects. Moreover, the findings are limited to animal models, and extrapolation to human physiology should be done with caution.

Future studies should investigate the long-term reproductive outcomes, including fertility rates and offspring viability, and explore the mechanistic pathways involved, such as NF-κB signaling and PPAR activation. Dose-response studies and combined therapy regimens may further elucidate optimal prote-ctive strategies.

In conclusion, while both rosuvastatin and ramipril offer antioxidant-based protection against doxorubicin-induced testicular injury, ramipril appears to confer greater functional benefits, especially in preserving hormonal balance and sperm motility. These results provide a compelling rationale for further translational research on ACEIs as fertility-protective agents in oncology settings.

## AUTHORS’ CONTRIBUTIONS

BC: Supervision, conceptualization, methodology, review and editing, and manuscript drafting. BA: Initial draft, performed the experiments, and collected and analyzed data. SH: Study design, review, editing, and analysis of data. SG: Study design, review, and editing. GK: experimental and data analysis, review, and editing. SGK: Review, analysis, and editing. All authors have read and approved the final manuscript.
